# Calcipotriol and betamethasone dipropionate synergistically enhances the balance between regulatory and proinflammatory T cells in a murine psoriasis model

**DOI:** 10.1038/s41598-019-52892-1

**Published:** 2019-11-08

**Authors:** Kyosuke Satake, Toru Amano, Tadao Okamoto

**Affiliations:** 1 R&D division, Kyowa Kirin Co., Ltd, 1188 Shimotogari, Nagaizumi-cho, Sunto-gun, Shizuoka, 411-8731 Japan; 2 Medical Affairs Department, LEO Pharma K.K., 1-105, Kanda Jimbocho, Chiyoda-ku, Tokyo, 101-0051 Japan

**Keywords:** Pathogenesis, Immunology

## Abstract

A topical medication combining calcipotriol (Cal) and betamethasone dipropionate (BDP) has proven effective in a number of randomized controlled trials performed in patients with psoriasis, but its mechanism of action has not been fully elucidated. We investigated whether the combination of Cal and BDP (Cal/BDP) in this topical medication had a synergistic effect on psoriasis-like dermatitis and explored the underlying immunological mechanisms in a murine psoriasis model induced by application of imiquimod. Cal/BDP synergistically inhibited ear thickening induced by imiquimod compared to monotherapy with either Cal or BDP. In addition, Cal/BDP significantly suppressed the interleukin (IL)-23/IL-17-producing T (T17) pathogenic axis, including expression of *IL-17a*, *IL-23a*, *IL-22* and *TNF-α* mRNA in skin lesions and expansion of CCR6^+^ γδ T17 cells in the draining lymph nodes. Notably, Cal/BDP synergistically induced regulatory CD8^+^ T cells and also improved the balance between regulatory CD8^+^ or CD4^+^ T cells and proinflammatory CCR6^+^ γδ T17 cells in the draining lymph nodes. These results suggest synergistic anti-psoriatic activity of Cal/BDP with normalization of the imbalance between regulatory CD8^+^ or CD4^+^ T cells and proinflammatory CCR6^+^ γδ T17 cells, which contributes to successful control of psoriasis by Cal-BDP combination therapy.

## Introduction

Psoriasis is one of the most common immune-mediated chronic inflammatory skin diseases, being characterized by hyperproliferation and abnormal differentiation of epidermal keratinocytes as well as the infiltration of inflammatory cells into skin lesions. Various immune abnormalities have been suggested to be involved in the pathogenesis of psoriasis. The activation and accumulation of CD4^+^ T helper 17 cells (Th17 cells), interleukin (IL)-17-producing CD8^+^ T cells (Tc17 cells), IL-17-producing γδ T cells (γδ T17 cells), and tumor necrosis factor-α/inducible nitric oxide synthase-producing dendritic cells (TIP-DCs) can be observed in psoriatic lesions, and these immune cells produce TNF-α, IL-23, and IL-17^1,2^. Importantly, IL-23 produced by TIP-DCs is essential for the proliferation and survival of IL-17-producing T cells (T17 cells) including Th17, Tc17 and γδ T17 cells^3,4^. In addition, it has been demonstrated that biological agents such as anti-IL-17A antibody, anti-IL-17 receptor A antibody, and anti-IL-23p19 antibody provide effective treatment for psoriasis^3^. Accordingly, the “IL-23-T17 pathogenic axis” is thought to play an important role in psoriasis.

Recent studies have suggested that an imbalance between regulatory CD4^+^CD25^+^Foxp3^+^ T cells (CD4^+^ Tregs) and Th17 cells is important in the pathogenesis of psoriasis^5^, while CD4^+^ Treg dysfunction has also been detected in this disease^6^. Other regulatory cell subsets have been described, such as regulatory CD8^+^CD122^+^ T cells (CD8^+^ Tregs)^7,8^, and regulatory CD1d^+^CD5^+^IL-10^+^ B cells (Bregs) also exist^9^. CD8^+^ Tregs are reported to play an important immunosuppressive role^10^, with human CD8^+^CXCR3^+^ T cells being the counterpart of murine CD8^+^ Tregs^11,12^. It was also reported that Bregs are decreased in number and show functional impairment in psoriasis patients^13^. Thus, it has been suggested that the successful treatment for psoriasis may require correcting the imbalance between regulatory T/B cells and proinflammatory T cells.

The topical combination of the active vitamin D_3_ [1,25-dihydroxychlecalciferol; 1,25(OH)_2_D_3_ or VD_3_] analogue Calcipotriol (Cal) and the corticosteroid Betamethasone dipropionate (BDP) is widely used as the mainstay for treating psoriasis^14^. Treatment with Cal-BDP combination therapy is more effective than either Cal or BDP monotherapy^15^. Corticosteroids are potent anti-inflammatory agents that block several inflammatory pathways^16^ and induce apoptosis of inflammatory cells^17^, while VD_3_ analogues promote the differentiation and inhibit the proliferation of keratinocytes, as well as suppressing cytokine production by T cells, dendritic cells, and keratinocytes^18^. An examination of skin biopsy samples from psoriasis patients has shown that Cal-BDP combination therapy inhibits epidermal thickening and T17 cell infiltration more strongly than Cal or BDP monotherapy^19^. These studies have suggested that Cal-BDP combination therapy suppresses the IL-23/T17 pathogenic axis. However, whether Cal and BDP have a synergistic effect on psoriasis *in vivo* remains unclear.

It has also been suggested that Cal-BDP combination therapy is the most useful topical option for long-term management and maintenance in the treatment of psoriasis^20,21^. However, rebound of psoriasis sometimes occurs after stopping corticosteroid treatment. VD_3_ analogues exert their immunomodulatory effect by enhancing the immunosuppressive activity of CD4^+^ Tregs^22^ and show more persistent action against psoriasis than BDP^23^. In addition, Cal-BDP combination therapy is reported to be more useful for the long-term management of psoriasis than Cal monotherapy^20^. Thus, different mechanisms may be involved in the long-term effects of these agents, perhaps involving the induction of specific and potent regulatory immune cells by Cal-BDP combination therapy. However, the effect of this therapy on CD4^+^ Treg, CD8^+^ Treg, and Breg cells has not been systematically explored.

In this study, we investigated whether or not Cal and BDP (Cal/BDP) had a synergistic effect on imiquimod (IMQ)-induced psoriasis-like dermatitis and explored the immunological mechanisms underlying the actions of Cal/BDP.

## Results

### Synergistic effect of topical Cal/BDP on IMQ-induced psoriasis-like dermatitis in mice

Consistent with a previous report^24^, the topical application of IMQ to the ear for 6 consecutive days induced psoriasis-like lesions that featured scaling, skin thickening, and erythema (data not shown). Thus, this demonstrates many features of human psoriasis.

**Figure 1:**
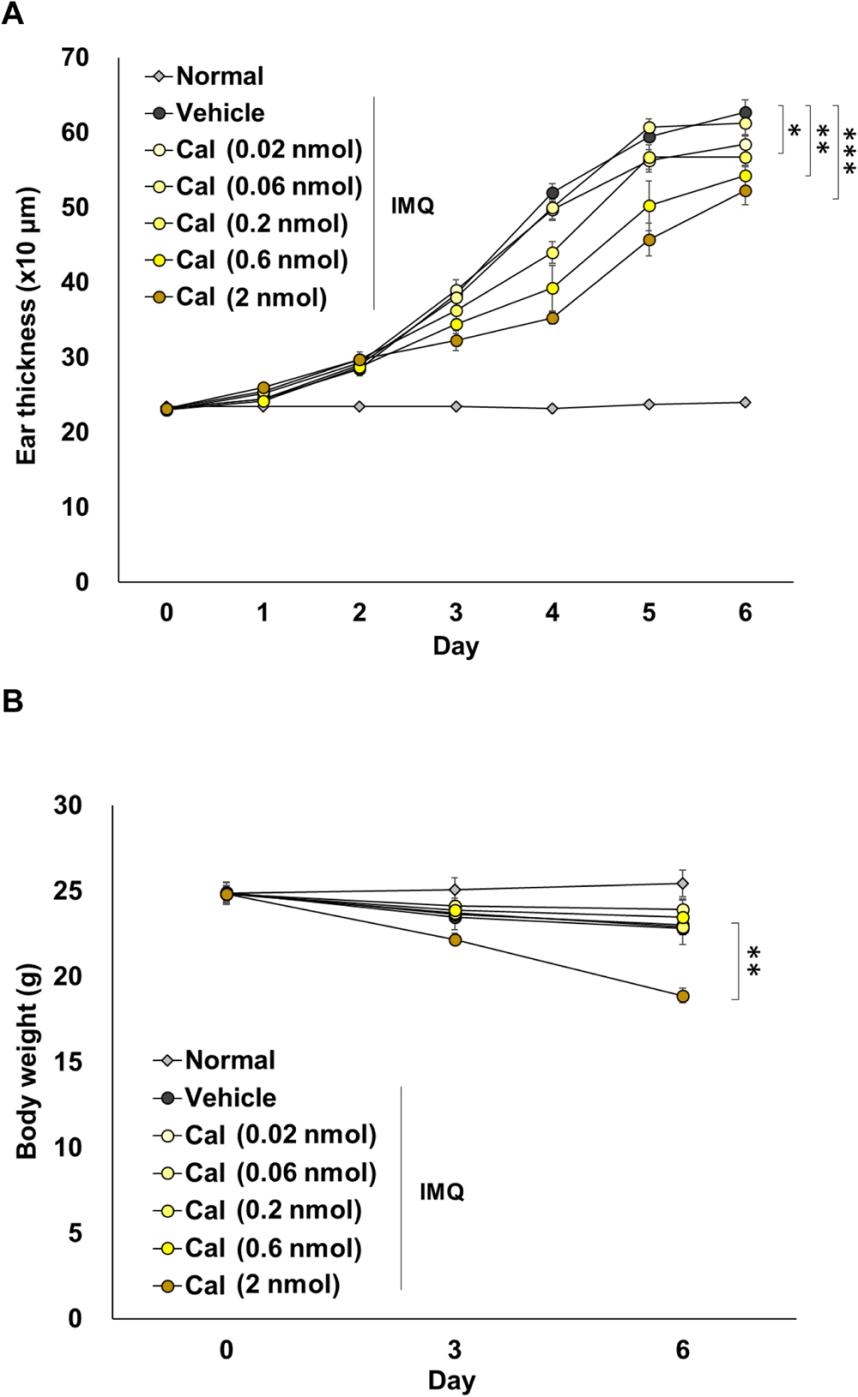
The optimal dose of topical Cal in mice with IMQ-induced psoriasis-like dermatitis. Mice received topical application of IMQ cream on the ear for 6 consecutive days, and were treated with 0.02, 0.06, 0.2, 0.6, and 2 nmol of Cal 1 h before IMQ application. Normal mice were used as a negative control. (**A**) Ear thickness was measured every day. (**B**) Body weight was measured every three days during the experiment. The values and vertical bar represent the mean ± SE of 4 mice. *p < 0.05, **p < 0.01, ***p < 0.001 vs. vehicle group by Dunnett test among the vehicle, Cal, BDP, and Cal/BDP groups.

First, we examined the optimum dose of topical Cal (0.02–2.0 nmol) for treating IMQ-induced psoriasis-like dermatitis in mice, because the structure of mouse skin differs from that of human skin (e.g. the epidermis has a few layers in mice vs. about 10 layers in humans). Topical Cal (0.2–2.0 nmol) significantly suppressed the IMQ-induced ear thickness in a dose-dependent manner (Fig. 1A). When Cal was applied at 2.0 nmol for 6 consecutive days, ear thickness showed maximal and significant reduction, but significant weight loss was observed (Fig. 1B, p < 0.01 vs. vehicle). Therefore, the dose of Cal at 0.6 nmol, which was effective without causing weight loss, was set when examining its combined effect with BDP.

**Figure 2:**
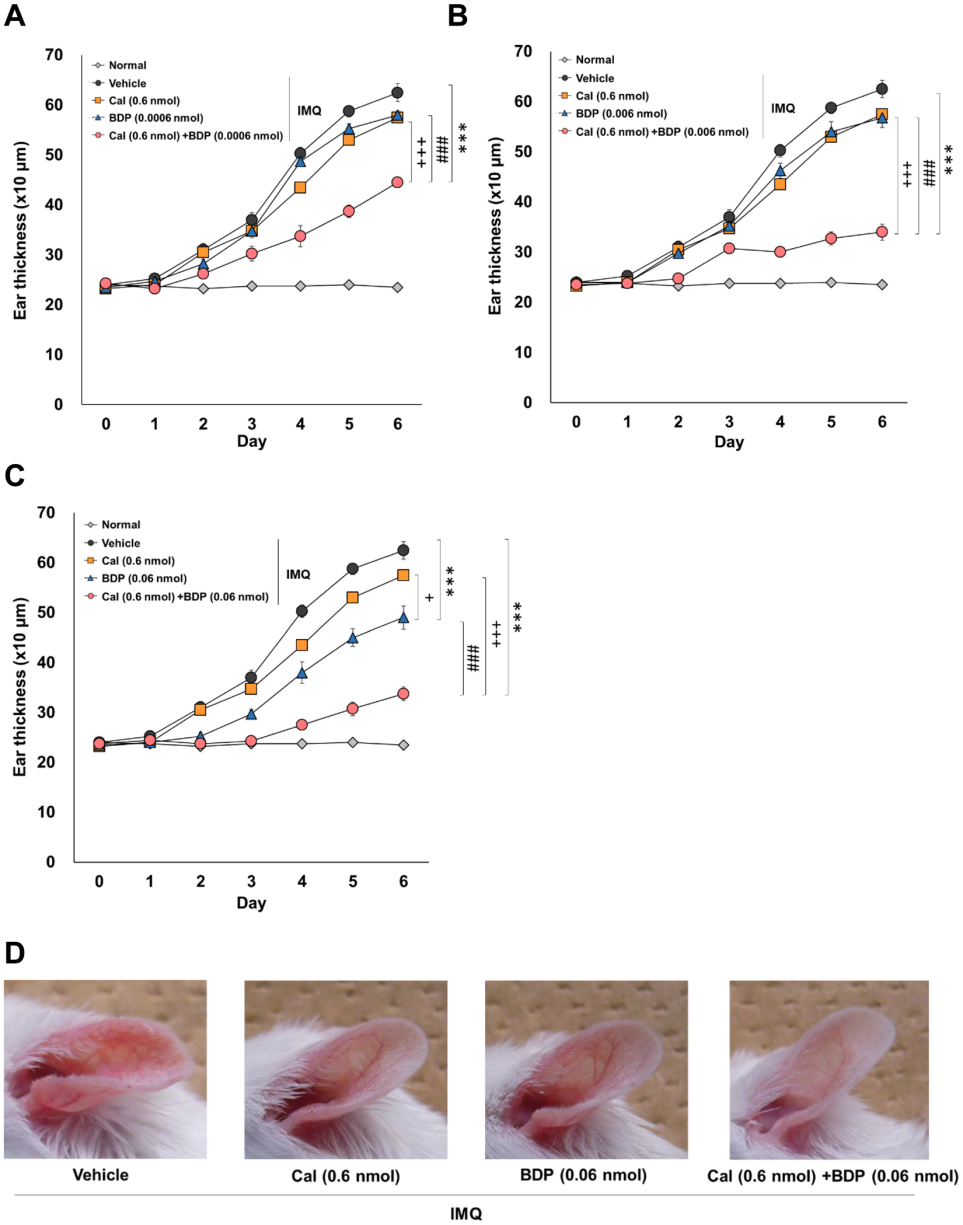
The optimal dose of topical BDP in combination with topical Cal in mice with IMQ-induced psoriasis-like dermatitis. Mice received the topical application of IMQ cream on the ear for 6 consecutive days and were treated with 0.6 nmol of Cal, 0.0006 (**A**), 0.006 (**B**), and 0.06 nmol (**C**) of BDP or the combination of Cal and BDP 1 h before IMQ application. Representative gross appearances of skin lesions one day after the final IMQ application (**D**). Normal mice were used as a negative control. Ear thickness was measured every day. The values and vertical bar represent the mean ± SE of 4 mice. ***p < 0.001 vs. vehicle group, ^+^p < 0.05, ^+++^p < 0.001 vs. Cal group, ^###^p < 0.001 vs. BDP group by Tukey test among the vehicle, Cal, BDP, and Cal/BDP groups.

Next, we examined the optimum dose of BDP (0.0006–0.06 nmol) combined with Cal at 0.6 nmol. As shown in Fig. 2, The combination of Cal and BDP showed a dose-dependent synergistic effect at any dose of BDP [8.7%, 27.6%, and 85.2% reduction in ear thickness by 0.6 nmol Cal, 0.06 nmol BDP, and the combination of Cal and BDP (0.6 nmol/0.06 nmol), respectively (Fig. 2C,D)]. The combinations of 0.6 nmol Cal and 0.06 nmol BDP were therefore selected for use in further experiments to explore its immunological mechanisms.

### Inhibition of the expression of cytokines related to the IL-23/T17 pathogenic axis in psoriatic lesions by Cal and Cal/BDP, but not BDP alone

**Figure 3:**
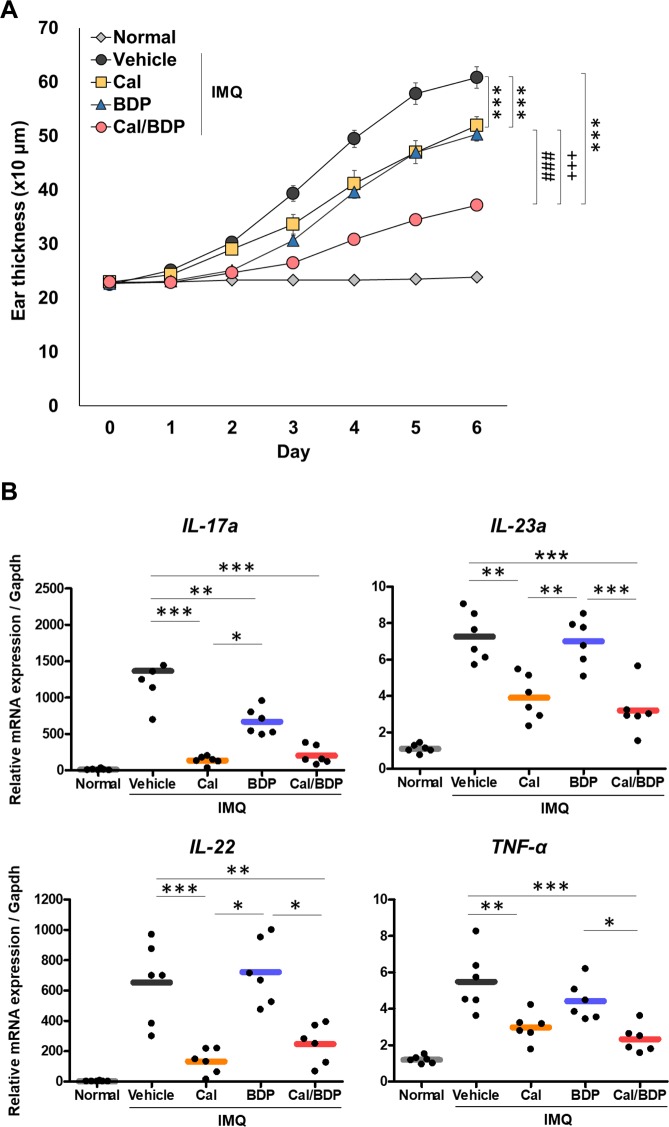
Synergistic effects of the topical combination of Cal and BDP on IMQ-induced psoriasis-like dermatitis in mice through suppressing the expression of cytokines related to the IL-23/T17 pathogenic axis. Mice received the topical application of IMQ cream on the ear for 6 consecutive days and were treated with 0.6 nmol Cal, 0.06 nmol BDP, or the combination of 0.6 nmol Cal and 0.06 nmol BDP 1 h before each IMQ application. (**A**) Effects of Cal, BDP, and Cal/BDP on ear thickness. Ear thickness was measured every day. The values and vertical bar represent the mean ± SE of 6 mice. ***p < 0.001 vs. vehicle group, ^+++^p < 0.001 vs. Cal group, ^###^p < 0.001 vs. BDP group by Tukey test among the vehicle, Cal, BDP, and Cal/BDP groups. (**B**) Inhibition of the expression of cytokines related to the IL-23/T17 pathogenic axis in IMQ-induced skin lesion. The expression in IMQ-induced skin lesions of the indicated mRNAs, which are related to the IL-23/T17 pathogenic axis, was analyzed by quantitative real-time RT-PCR. The expression is relative to the housekeeping gene Gapdh. Each symbol represents an individual mouse, and the horizontal lines indicate the means of the groups (n = 6). Similar results were obtained in two independent experiments. *p < 0.05, **p < 0.01, ***p < 0.001 by Tukey test among the vehicle, Cal, BDP, and Cal/BDP groups.

Cal/BDP (0.6 nmol Cal and 0.06 nmol BDP) showed a synergistic effect on the IMQ-induced ear thickness with good reproducibility (Fig. 3A, p < 0.001 vs. vehicle). In contrast, monotherapy with either Cal or BDP only slightly but significantly reduced the ear thickness (Fig. 3A, p < 0.001 for Cal and BDP groups vs. vehicle).

Next, we examined whether topical Cal, BDP, or Cal/BDP inhibited the expression of cytokine genes (*IL-17a*, *IL-22*, *IL-23a*, and *TNF-α*) related to the IL-23/T17 pathogenic axis in psoriatic skin lesions on day 7 of IMQ treatment. Reverse transcription polymerase chain reaction (RT-PCR) revealed that the *IL-17a* mRNA expression was induced in IMQ-treated ear skin, while topical treatments with Cal, BDP, or Cal/BDP suppressed the *IL-17a* upregulation in the skin lesions (Fig. 3B). The suppressive effect of Cal or Cal/BDP (p < 0.001 vs. vehicle) was significantly stronger than that of BDP alone (p < 0.01 vs. vehicle) (Fig. 3B). RT-PCR also revealed that expression of mRNA for *IL-22, IL-23a*, and *TNF-α* was markedly increased in IMQ-treated ear skin (Fig. 3B). In correspondence with the attenuation of the *IL-17a* mRNA expression, the IMQ-induced upregulation of *IL-23a*, *IL-22*, and *TNF-α* mRNA expression was significantly suppressed by Cal or Cal/BDP but not by BDP alone. IL-23 is known as a “master regulator” of proinflammatory T17 cell development because it has a critical role in the proliferation and maintenance of proinflammatory T17 cells^3^. Therefore, it is noteworthy that Cal and Cal/BDP significantly suppressed the *IL-23a* mRNA expression while BDP did not. Similar findings were also obtained for the *IL-22* mRNA expression.

These findings suggest that Cal and Cal/BDP suppressed the IL-23/T17 pathogenic axis in psoriatic lesions.

### Inhibition of CCR6^+^ γδ T17 cell expansion in draining lymph nodes (DLNs) by Cal/BDP

In the present murine model of IMQ-induced psoriasis, proinflammatory γδ T17 cells migrate from inflamed skin lesions to dLNs where these cells undergo marked expansion before homing back to the skin lesions, thus contributing to the exacerbation of psoriasis-like dermatitis^25^. It was reported that the topical application of Cal inhibits the T17 cell accumulation in both the treated skin lesions and their dLNs, leading to improvement of psoriasis-like dermatitis^26^. Therefore, we used flow cytometry to analyze the effect of Cal, BDP, and Cal/BDP on T cell subsets in the dLNs on day 7 (1 day after the end of 6-day IMQ treatment).

**Figure 4:**
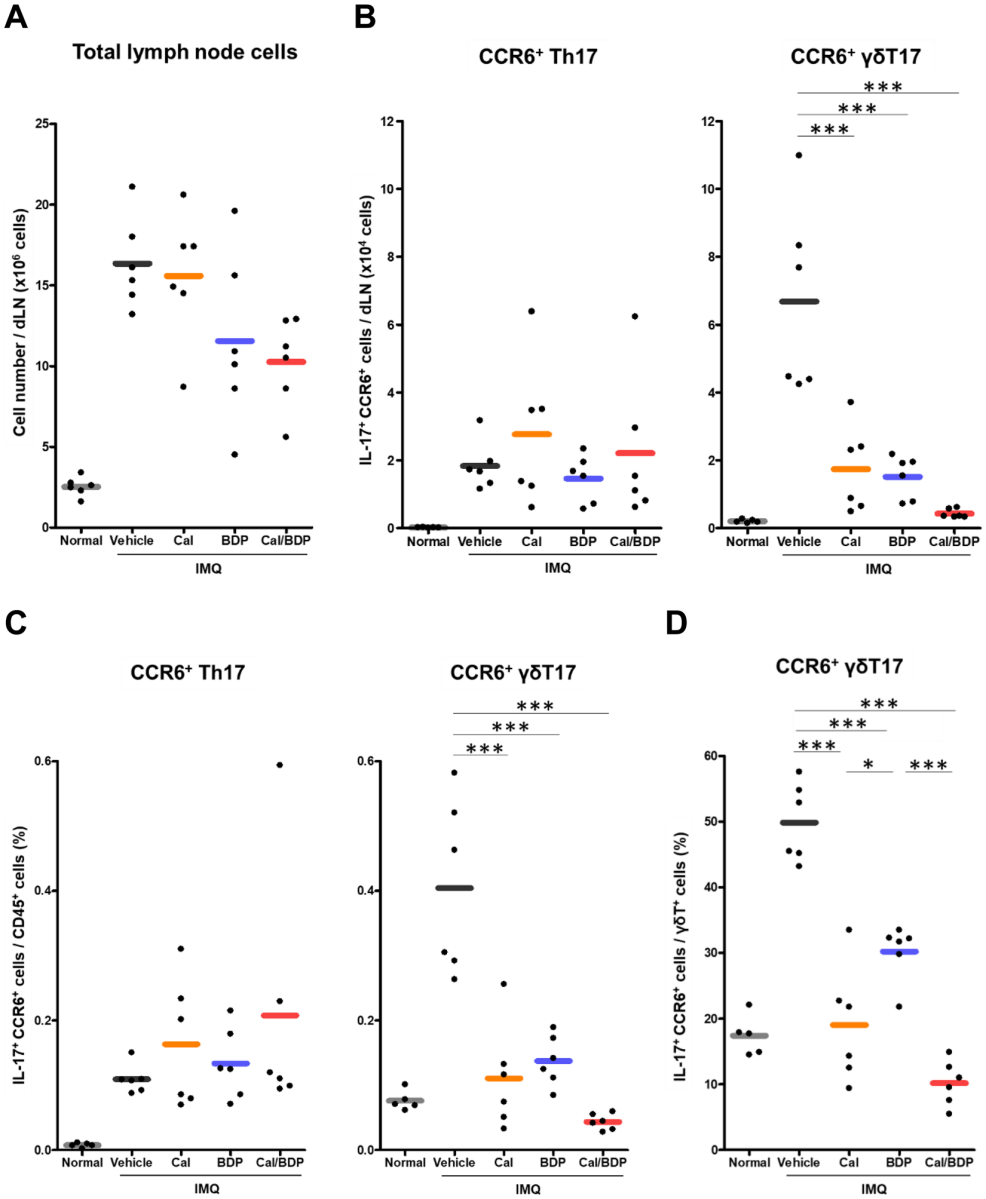
The inhibition of the CCR6^+^ γδT17 cell expansion in dLNs by Cal/BDP as the therapeutic target. Mice received the topical application of IMQ cream on the ear for 6 consecutive days and were treated with 0.6 nmol Cal, 0.06 nmol BDP, or the combination of 0.6 nmol Cal and 0.06 nmol BDP 1 h before each IMQ application. (**A**) Reduction in the absolute number of lymph node cells in an individual dLN by BDP and Cal/BDP. Cells from the individual dLN were isolated 1 day after the final IMQ application, and each total lymph node cell number was counted by CDA-1000. (**B**) The inhibition of the expansion of CCR6^+^ γδT17, but not CCR6^+^ Th17, by Cal, BDP, and Cal/BDP in dLNs. Cells from the individual dLN were isolated 1 day after the final IMQ application, and each total lymph node cell number was counted using a CDA-1000. The rates of CCR6^+^ Th17 and CCR6^+^ γδT17 in the CD45^+^ leukocyte population were analyzed by flow cytometry. The numbers of each cell subset were calculated by multiplying the total lymph node cell numbers by the ratio of each cell subset. (**C**) Reduction in the proportions of CCR6^+^ γδT17, but not CCR6^+^ Th17, in the CD45^+^ leukocyte population by Cal, BDP, and Cal/BDP in dLNs. The rates of CCR6^+^ Th17 and CCR6^+^ γδT17 in the CD45^+^ leukocyte population were analyzed by flow cytometry 1 day after the final IMQ application. (**D**) Reduction in the proportion of CCR6^+^ γδT17 in the γδT cell subset by Cal, BDP, and Cal/BDP in dLNs. Rates of CCR6^+^ γδT17 in the γδT cell subset were analyzed by flow cytometry 1 day after the final IMQ application. Each symbol represents an individual mouse, and the horizontal lines indicate the means of the groups (n = 5–6). *p < 0.05, ***p < 0.001 by Tukey test among the vehicle, Cal, BDP, and Cal/BDP groups.

First, we examined the effect of topical Cal, BDP, or Cal/BDP on the total cell count in dLNs using a cell counter in order to assess the nonspecific anti-inflammatory activity. The total cell count in dLNs was reduced by treatment with BDP or Cal/BDP compared to vehicle treatment but was not reduced by Cal (16.4 × 10^6^, 15.6 × 10^6^, 11.6 × 10^6^, and 10.3 × 10^6^ cells/dLNs in vehicle, Cal, BDP, and Cal/BDP, respectively) (Fig. 4A). This result suggests that corticosteroids, such as BDP, affect a wide variety of inflammatory cells in dLNs by suppressing the expansion or recruitment of such cells.

IL-17 has a critical role in the pathogenesis of psoriasis, and the major cells producing IL-17 in psoriatic skin lesions are thought to be Th17^27^ and γδ T17 cells^28^. In a murine model of IMQ-induced psoriasis, IL-23 was reported to induce γδ T17 cells in dLNs, which are the major IL-17 producers and express the chemokine receptor CCR6 (CCR6^+^ γδ T17 cells). Thus, CCR6^+^ γδ T17 cells are thought to play a pivotal role in the development of psoriasis-like lesions^29,30^. Accordingly, we next examined whether or not Cal/BDP suppressed the IMQ-induced expansion of proinflammatory CCR6^+^ T17 cells in dLNs more effectively than either Cal or BDP alone.

In our psoriasis model, both CCR6^+^ Th17 cells and CCR6^+^ γδ T17 cells showed expansion in the dLNs, but the total number of CCR6^+^ Th17 cells was much lower than that of CCR6^+^ γδ T17 cells (Fig. 4B), as previously reported^26^. A flow cytometric analysis also showed that IMQ treatment caused a marked increase in the percentage of CCR6^+^ γδ T17 cells (Fig. 4C) and a small increase in the percentage of CCR6^+^ Th17 cells (Fig. 4C) in the CD45^+^ subset, which is the leukocyte common antigen-positive cell population. These results suggest that CCR6^+^ γδ T17 cells are the major IL-17A-producing cell population in the dLNs in the present mouse model.

The total number of CCR6^+^ γδ T17 cells in the dLNs was significantly reduced by all 3 treatments (Fig. 4B, p < 0.001 vs. vehicle). However, Cal/BDP inhibited the expansion of CCR6^+^ γδ T17 cells more strongly than either of the single agents (Fig. 4B). In contrast, the total number of CCR6^+^ Th17 cells in the dLNs was not markedly influenced by any of the three treatments (Fig. 4B). Regarding changes in the percentage of proinflammatory CCR6^+^ T17 cells in the CD45^+^ subset, the accumulation of CCR6^+^ γδ T17 cells, but not CCR6^+^ Th17 cells, was most strongly attenuated by Cal/BDP (Fig. 4C). When the change in the percentage of CCR6^+^ γδ T17 cells in the γδ T subset was assessed, the reduction achieved by Cal/BDP was also significantly greater than that by BDP alone (p < 0.001) (Fig. 4D).

Thus, the suppression of CCR6^+^ γδ T17 cells by Cal, BDP, or Cal/BDP corresponded to the effects of these agents on psoriasis-like dermatitis (Fig. 3A). These results suggest that CCR6^+^ γδ T17 cells expanding in the dLNs are not only the main source of IL-17A but also represent a therapeutic target for psoriasis.

### Induction of CD8^+^ Tregs by Cal/BDP vs. suppression of the Foxp3 expression on CD4^+^ Tregs by BDP

There have been many reports that CD4^+^ Tregs are dysfunctional and undergo trans-differentiation into Th17 cells in severe psoriasis^6,31^. IL-23 strongly promotes trans-differentiation of CD4^+^ Tregs into Th17 cells^1^, which is associated with the loss of Foxp3 expression^31^. In contrast, topical Cal was reported to induce CD4^+^ Tregs in the dLNs^32^, and we found that Cal and Cal/BDP significantly suppressed the *IL-23a* expression in psoriasis-like skin lesions (Fig. 3B). Other subsets of regulatory cells, CD8^+^ Tregs and Bregs, were recently reported to also play a role in animal models of autoimmune diseases, such as colitis^7^ and psoriasis^33^. Therefore, we used flow cytometry to investigate the effects of Cal, BDP, and Cal/BDP on these 3 regulatory cell subsets in the dLNs on day 7 (1 day after the end of 6-day IMQ treatment).

**Figure 5:**
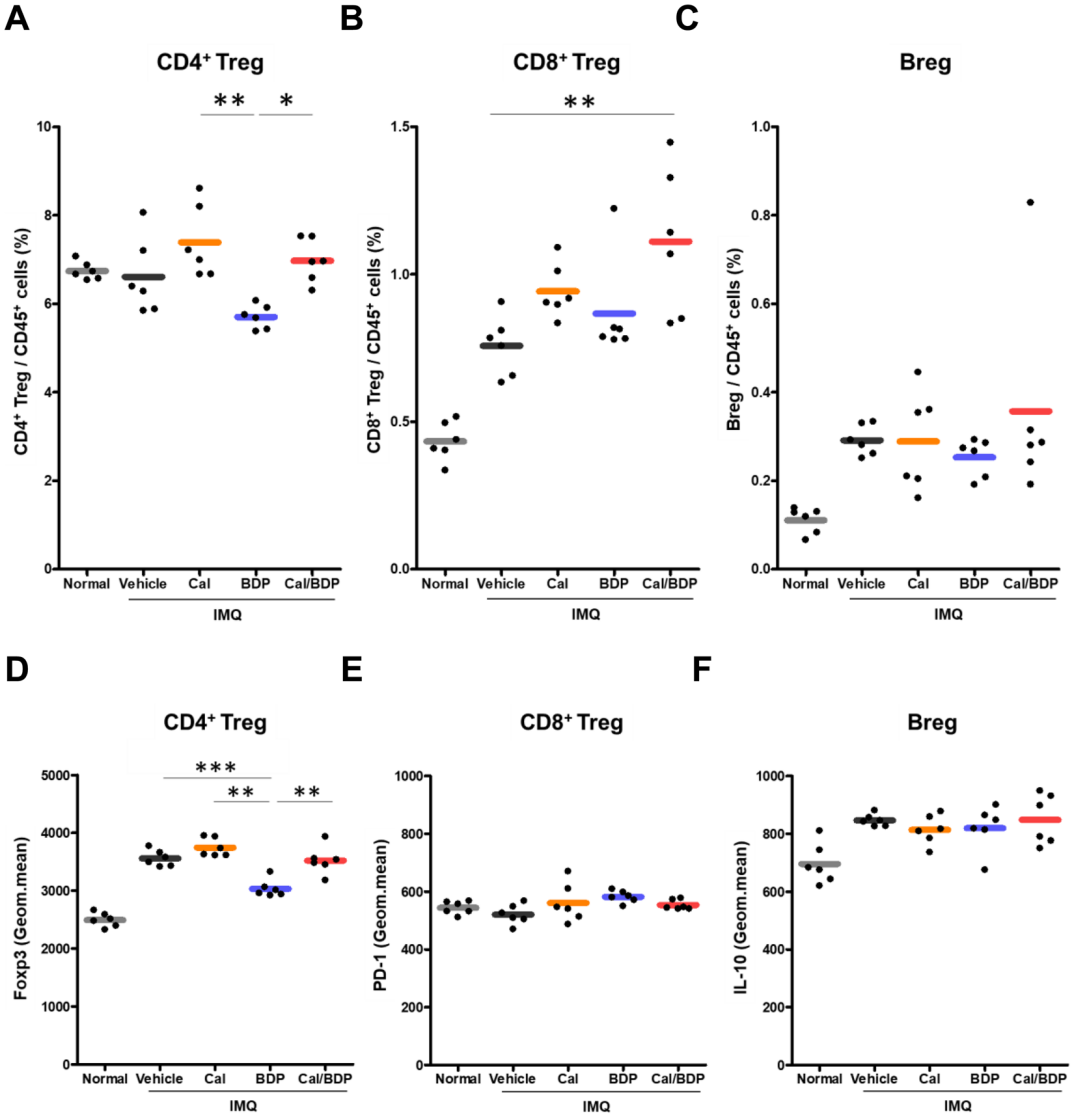
The induction of CD8^+^ Tregs by Cal/BDP and the suppression of the Foxp3 expression in CD4^+^ Tregs by BDP. Mice received the topical application of IMQ cream on the ear for 6 consecutive days and were treated with 0.6 nmol Cal, 0.06 nmol BDP, or the combination of 0.6 nmol Cal and 0.06 nmol BDP 1 h before each IMQ application. (**A–C**) Changes in the proportion of CD4^+^ Tregs (gated on CD45^+^CD3^+^CD4^+^CD25^+^Foxp3^+^), CD8^+^ Tregs (gated on CD45^+^CD3^+^CD8^+^CD122^+^PD-1^+^), and Bregs (gated on CD45^+^B220^+^CD5^+^CD1d^+^IL-10^+^) in the CD45^+^ leukocyte population. The rates of CD4^+^ Tregs, CD8^+^ Tregs, and Bregs in the CD45^+^ leukocyte population were analyzed by flow cytometry 1 day after the final IMQ application. (**D–F**) The expression of Foxp3 in the CD4^+^ Treg subset, PD-1 in the CD8^+^ Treg subset, and IL-10 in the Breg subset in dLNs. The geometric means of Foxp3 in the CD4^+^ Treg subset, of PD-1 in the CD8^+^ Treg subset, and of IL-10 in the Breg subset in dLNs were analyzed by flow cytometry 1 day after the final IMQ application. Each symbol represents an individual mouse, and the horizontal lines indicate the means of the groups (n = 6). *p < 0.05, **p < 0.01, ***p < 0.001 by Tukey test among the vehicle, Cal, BDP, and Cal/BDP groups.

We first examined the influence of topical Cal, BDP, and Cal/BDP on the percentages of CD4^+^ Tregs (CD4^+^CD25^+^Foxp3^+^), CD8^+^ Tregs (CD8^+^CD122^+^PD-1^+^), and Bregs (B220^+^CD1d^+^CD5^+^IL-10^+^) in the CD45^+^ leukocyte population of the dLNs. We found that the percentage of CD4^+^ Tregs was significantly lower after treatment with BDP than after treatment with Cal (p < 0.01) or Cal/BDP (p < 0.05) (Fig. 5A). Notably, the percentage of CD8^+^ Tregs was increased by Cal and significantly increased by Cal/BDP (p < 0.01) compared to the vehicle, while BDP had no effect (Fig. 5B). In contrast, the percentage of Bregs was not altered by any of the three treatments (Fig. 5C).

Next, we examined the effects of these three topical treatments on the level of Foxp3 protein expression by CD4^+^ Tregs in the dLNs, as well as PD-1 expression by CD8^+^ Tregs and IL-10 expression by Bregs (Fig. 5D–F). Of note, we found that the geometric mean Foxp3 expression by CD4^+^ Tregs was significantly suppressed in the BDP group compared with the Cal and Cal/BDP groups (Fig. 5D), suggesting that the immunosuppressive activity of CD4^+^ Tregs was inhibited by BDP.

### Restoration of the balance between regulatory CD8^+^ or CD4^+^ T cells and CCR6^+^ γδ T17 cells by Cal/BDP

It was recently reported that Cal-BDP combination therapy increases CD4^+^CD25^+^Foxp3^+^CD127^lo^ Tregs and reduces Th17 cells in the blood and skin lesions of patients with psoriasis, suggesting that Cal-BDP combination therapy restores the Treg-T17 balance^5^.

**Figure 6:**
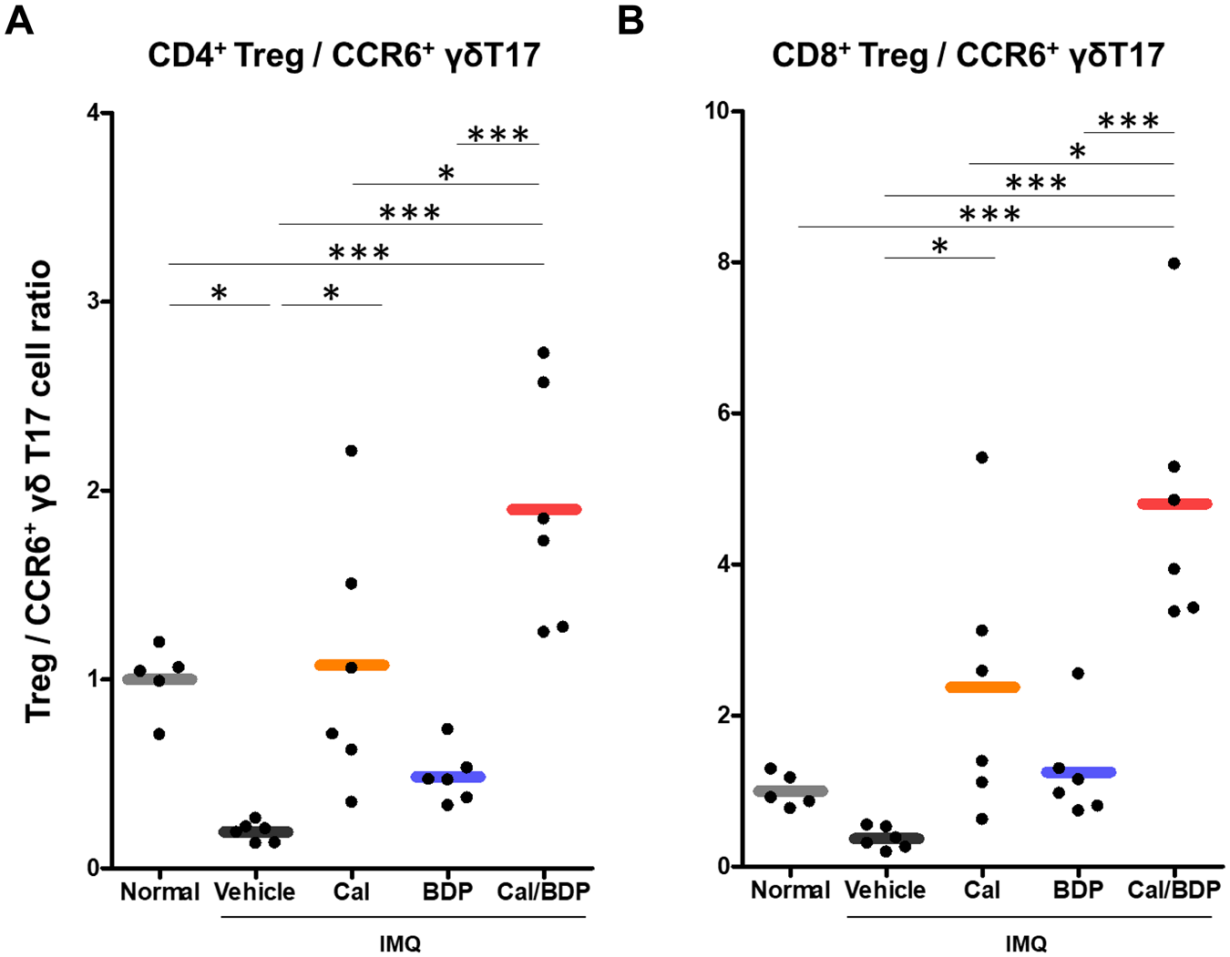
Restoration of the balance between Tregs and pathogenic γδT17 cells by treatment with Cal/BDP in CD45^+^ cells. Mice received the topical application of IMQ cream on the ear for 6 consecutive days and were treated with 0.6 nmol Cal, 0.06 nmol BDP, or the combination of 0.6 nmol Cal and 0.06 nmol BDP 1 h before each IMQ application. Cells from the individual dLN were isolated 1 day after the final IMQ application. The ratios of Tregs to pathogenic γδT17 cells were calculated by dividing the ratio of CD4^+^ Tregs (**A**) or CD8^+^ Tregs (**B**) by the ratio of CCR6^+^ γδT17 cells in CD45^+^ cells. Each symbol represents an individual mouse, and the horizontal lines indicate the means of the groups (n = 5–6). *p < 0.05, ***p < 0.001 by Tukey test among the normal, vehicle, Cal, BDP, and Cal/BDP groups.

Therefore, we examined the effects of topical Cal, BDP, and Cal/BDP on the balance between CD8^+^ or CD4^+^ regulatory T cells and proinflammatory T17 cells by determining the ratio of CD4^+^ Tregs to CCR6^+^ γδ T17 cells (CD4^+^ Treg/γδ T17 ratio) as well as the ratio of CD8^+^ Tregs to CCR6^+^ γδ T17 cells (CD8^+^ Treg/γδ T17 ratio), because CD4^+^ Tregs were suppressed by BDP and CD8^+^ Tregs were induced by Cal/BDP. As shown in Fig. 6A,B, IMQ treatment significantly reduced the CD4^+^ Treg/γδ T17 ratio (0.2) compared with that in normal mice (1.0) and also reduced the CD8^+^ Treg/γδ T17 ratio (0.4), albeit not significantly. Cal significantly restored these ratios to equal or greater than normal. In particular, Cal/BDP caused marked increases in the CD4^+^ Treg/γδ T17 ratio and CD8^+^ Treg/γδ T17 ratio by 1.9-fold (p < 0.001 vs. normal) and 4.8-fold (p < 0.001 vs. normal), respectively, compared with the ratios in normal mice. In contrast, BDP exerted no such effect, and the CD4^+^ Treg/γδ T17 ratio remained low.

## Discussion

This study provided the first evidence that topical Cal/BDP caused synergistic improvement of IMQ-induced psoriasis-like dermatitis *in vivo* while suppressing the expression of cytokines related to the IL-23/T17 pathogenic axis, such as *IL-17a* and *IL-23a*, in psoriatic lesions. In this study, we optimized the doses of Cal and BDP for our psoriasis model because the structures of mouse skin and human skin are considerably different.

In our model and in accordance with previous studies^26^, IMQ treatment led to the marked expansion of CCR6^+^ γδ T17 cells in the dLNs, and these cells were the major IL-17A-producing population. In humans, it was reported that γδ T17 cells infiltrate the dermis in psoriatic skin lesions and produce a substantial amount of IL-17^28^. Additionally, the levels of the local chemokine CCL20, a ligand for CCR6, were reported to be increased in psoriatic skin lesions along with CCR6 and IL-17A, thereby CCR6^+^ T17 cells are recruited to inflamed skin^34^. Interestingly, the efficacy of Cal, BDP, and Cal/BDP seemed to be directly proportional to the percent reduction of CCR6^+^ γδ T17 cells by these agents. This suggests that the treatment may target CCR6^+^ γδ T17 cells expansion in the dLNs in mice with IMQ-induced psoriasis-like dermatitis. In addition, we found that Ca/BDP significantly attenuated the expansion of these cells compared with either Cal or BDP alone. These findings are consistent with those of recent reports suggesting that the therapeutic target of Cal-BDP combination therapy is the IL-23/T17 pathogenic axis, with Cal/BDP suppressing the infiltration of T17 cells into the skin lesions^19^ and reducing the levels of proinflammatory cytokines *ex vivo*^18^.

The present study also demonstrated that the *IL-23a* mRNA expression in skin lesions of the mice was markedly suppressed by treatment with Cal or Cal/BDP. Several studies have shown that IL-23 is responsible for the trans-differentiation of CD4^+^ Tregs into Th17 cells^1^, in association with the loss of Foxp3 expression^35^. Conversely, treatment with IL-23p19 antagonists achieves a relatively long-term response in psoriasis patients, which can be partly explained by these agents promoting trans-differentiation of Th17 cells into CD4^+^ Tregs by blocking the action of IL-23^1^. Therefore, the suppression of IL-23 by Cal and Cal/BDP may contribute to correcting the imbalance between CD4^+^ Tregs and proinflammatory T17 cells in the dLNs, leading to a long-term clinical response of psoriasis. In contrast to Cal/BDP, treatment with BDP alone did not suppress the *IL-23a* mRNA expression. In addition, BDP significantly decreased the percentage of CD4^+^ Tregs and also suppressed the Foxp3 protein expression by CD4^+^ Tregs in the dLNs. These results suggest that BDP cannot restore the number of CD4^+^ Tregs or improve the function of these cells, as Foxp3 is required for the growth and proper functioning of CD4^+^ Tregs^36,37^. This may also explain why rebound of psoriasis sometimes occurs after stopping corticosteroid therapy.

This study provides the first evidence that treatment with Cal/BDP significantly induces CD8^+^ Tregs in the dLNs, and this induction of CD8^+^ Tregs may be associated with the marked improvement of ear thickening induced by IMQ in mice. CD8^+^ Tregs have been reported to play an essential role in suppressing various experimental autoimmune diseases^7,8,38^ and show stronger regulatory/immunosuppressive activity than CD4^+^ Tregs^11^. Human CD8^+^CXCR3^+^ T cells are the counterpart of murine CD8^+^ Tregs^11,12^. Considering that Bregs show an impaired function^13^ along with CD4^+^ Tregs in psoriasis^6^ and that CD4^+^ Tregs easily undergo trans-differentiation into Th17 cells^31^, the induction of CD8^+^ Tregs may be the key to the successful long-term management of this disease. However, the mechanisms underlying the induction of CD8^+^ Tregs by Cal/BDP have not been clarified. Thus, further studies are needed to determine the role of CD8^+^ Tregs induced by Cal/BDP in the long-term control of psoriasis, especially in the clinical setting. Although the relationship between CD8^+^ Tregs and CD4^+^ Tregs has not been clarified, the interaction seems to be fundamental to the prevention and cure of autoimmune diseases such as psoriasis.

Our results indicate that topical Cal/BDP shows a synergistic effect on psoriasis-like dermatitis *in vivo*, while suppressing the expression of cytokines related to the IL-23/T17 pathogenic axis and inhibiting expansion of proinflammatory T17 cells in the dLNs. The synergistic action of Cal/BDP enhances the balance between regulatory CD8^+^ or CD4^+^ T cells and proinflammatory T17 cells, as well as induces CD8^+^ Tregs. The normalization of both imbalances may contribute to the successful long-term control of psoriasis with Cal-BDP combination therapy.

## Materials and Methods

### Psoriasis model

BALB/c mice were treated topically with 5 mg of IMQ-containing cream (5%; Beselna Cream; Mochida Pharmaceutical, Tokyo, Japan) on the ventral side of both ears for 6 consecutive days. For treatment of IMQ-induced dermatitis, mice were treated daily with Cal, BDP, or the combination of Cal and BDP (Cal/BDP) dissolved in 20 μL of ethanol on the ear 1 h before IMQ application. During the treatment, the ear thickness was measured using a dial thickness gauge (OZAKI MFG., Tokyo, Japan) before the Cal, BDP, or Cal/BDP application every day under isoflurane anesthesia. Normal mice were used as a negative control. Body weight was measured every three days during the experiment. One day after the final IMQ application, after euthanasia, the right ear was excised and stored in RNAlater (QIAGEN, Tokyo, Japan) until RNA extraction, and dLNs were excised.

### Mice

Male BALB/c mice at six weeks of age were purchased from CLEA Japan (Tokyo, Japan) and acclimatized for at least one week. The mice were kept in a specific-pathogen-free animal facility with a maintained temperature of 19–25 °C, humidity of 30–70% and 12-h day/night cycle and given access to food and water *ad libitum*. The experiments were conducted in accordance with the Guiding Principles for the Care and Use of Laboratory Animals, and the experimental protocol used in this study was approved by the Committee for Animal Experiments at Kyowa Kirin Co., Ltd. (Shizuoka, Japan).

### Reagents

Calcipotriol was purchased from TocrisBioscience (Bristol, United Kingdom). Betamethasone dipropionate 17,21-dipropionate was purchased from Sigma-Aldrich Japan (Tokyo, Japan). Calcipotriol and Betamethasone dipropionate 17,21-dipropionate were dissolved in ethanol.

### Real-time reverse transcription polymerase chain reaction (RT-PCR)

Prior to the RNA isolation, the ear skin samples were homogenized with a TissueLyser (QIAGEN). RNA was obtained from ear skin samples with an RNeasy Fibrous Tissue Mini Kit (QIAGEN), and complementary DNA (cDNA) was synthesized using the SuperScript VILO cDNA Synthesis Kit (Invitrogen Japan, Tokyo, Japan). cDNA, probes, and TaqMan Gene Expression Master Mix (Applied Biosystems, Tokyo, Japan) were then mixed in a 96-well PCR plate, and quantitative PCR was performed using an ABI StepOnePlus^TM^ Real-Time PCR System (Applied Biosystems). The following Taqman Gene Expression Assay probes were used in this study: Gapdh: Mm99999915_g1, *Il17a*: Mm00439619_m1, *IL22*: Mm00444241_m1, *Il23a*: m00518984_m1 and *Tnf*: MM00443258_m1. Quantification of RNA was determined by comparing the threshold cycle number of each gene to the housekeeping gene Gapdh using the 2-ΔΔCT method.

### Antibodies

PerCp-Cy5.5 CD45 (104), APC-Cy7 CD3 (145-2C11), BV421 CCR6 (29-2L17), BV421 CD25 (PC61), APC-Cy7 B220 (RA-3-6B2), APC γδTCR (GL3), and FITC PD-1 (29 F.1A12) were purchased from Biolegend (San Diego, Calif). PE-Cy7 CD4 (RM4-5), APC CD5 (53–7.3), FITC CD1d (1B1), BV421 CD8 (53–6.7), and PE CD122 (TM-b1) were purchased from BD Biosciences (San Diego, Calif). APC Foxp3 (FJK-16s), PE IL-17A (eBio17B7), and PE IL-10 (JESS-16E3) were purchased from eBioscience (San Diego, Calif).

### Flow cytometry

Single-cell suspensions from dLNs were generated by gentle dissection using a glass slide in RPMI medium for a flow cytometric analysis. Total cell numbers in dLNs were counted by a CDA-1000 (Sysmex, Hyogo, Japan). For staining of surface markers, cells were suspended with the mixed solution of antibodies and incubated for 20 min at 4 °C protected from light. For intracellular staining of Foxp3, a Foxp3/Transcription Factor Staining Buffer Set (eBioscience) was used according to the manufacturer’s instructions. For intracellular staining of IL-17, single-cell suspensions from dLNs (1 × 10^7^ cells/mL) were stimulated with 50 ng/mL phorbol 12-myristate 13-acetate (Sigma-Aldrich) and 1 μg/mL ionomycin (Wako, Osaka, Japan) in the presence of Goldistop (BD Biosciences) for 5 h at 37 °C before surface staining. For intracellular staining of IL-10, single-cell suspensions from dLNs were stimulated with 10 μg/mL Lipopolysaccharides (Sigma-Aldrich), 50 ng/mL phorbol 12-myristate 13-acetate, and 500 ng/mL ionomycin in the presence of Goldistop for 5 h at 37 °C before surface staining. After surface staining, cells were fixed and permeabilized using Cytofix/Cytoperm Solution Kit (BD Biosciences). Permeabilized cells were suspended with the mixed solution of antibodies and incubated for 20 min at 4 °C protected from light. Each sample was analyzed on a BD FACSVerse^TM^ (BD Biosciences). The results were analyzed with the Flowjo software program (TreeStar, Ashland, OR). The numbers of each cell subset were calculated by means of the ratio of flow cytometry and total cell numbers in dLNs counted by CDA-1000. The ratios of Treg to pathogenic γδT17 cell were calculated by dividing the ratio of CD4^+^ Treg or CD8^+^ Treg by the ratio of CCR6^+^ γδT17 in CD45^+^ cells.

### Statistical analyses

Multiple comparisons among treatment groups were made using a one-way analysis of variance followed by Dunnett test or Tukey test with SAS software (SAS Institute, Cary, NC). A P value of < 0.05 was considered to be statistically significant.
